# Bloodstream Infections and Antibiotic Susceptibility Among Patients With Positive Blood Cultures at a Tertiary Care Hospital in Sri Lanka

**DOI:** 10.7759/cureus.108551

**Published:** 2026-05-09

**Authors:** Suneth Kumara, Maheswaran Umakanth, Sundaresan KT, Ayoma Thilakarathne, Nilanga Nishad, Shalomi Anopriyanth, Indika Gunawardana, Vaithehi Francis, Pakkiyaretnam Mayurathan

**Affiliations:** 1 General Medicine, Teaching Hospital Batticaloa, Batticaloa, LKA; 2 Clinical Sciences, Faculty of Health-Care Sciences, Eastern University of Sri Lanka, Batticaloa, LKA; 3 Anesthesia, Base Hospital, Kahawatta, LKA; 4 Gastroenterology and Hepatology, Sheffield Teaching Hospitals NHS Foundation Trust, Sheffield, GBR; 5 Medicine, Teaching Hospital Batticaloa, Batticaloa, LKA; 6 Community Medicine, Ministry of Health, Colombo, LKA; 7 Pathophysiology, Faculty of Health-Care Sciences, Eastern University of Sri Lanka, Batticaloa, LKA

**Keywords:** antibiotic susceptibility, bloodstream infection (bsi), qsofa, sepsis, sri lanka

## Abstract

Introduction: Bloodstream infections (BSIs) are a major cause of sepsis and in-hospital mortality. Context-specific data on antimicrobial resistance are essential for guiding empiric therapy.

Methods: A retrospective study was performed among 100 patients with clinically significant positive blood cultures admitted to the medical wards of Teaching Hospital Batticaloa, Sri Lanka, from January 1 to June 30, 2022. Demographic, clinical, microbiological, and outcome data were extracted from patient records. Associations between selected variables and clinical outcome were analyzed using the Statistical Package for Social Sciences version 26 (IBM Corp., Armonk, NY).

Results: The mean age was 53.6 years (SD = 16.31), and 82 (82%) had comorbidities, mainly diabetes mellitus. Coliforms caused 54 (54%) BSIs, and *Staphylococcus aureus* caused 19 (19%), of which 10/19 (52.6%) were methicillin-resistant *Staphylococcus aureus*. Urosepsis was the commonest focus of coliform bacteremia, while central line infection was the leading focus in *S. aureus* bacteremia. Among isolates tested, Coliforms remained susceptible to amikacin (34/35; 97.1%) and carbapenems (30/31; 96.8%), with low susceptibility to the third- and fourth-generation cephalosporins. *S. aureus* isolates showed good susceptibility to trimethoprim-sulfamethoxazole and clindamycin, and all isolates tested against penicillin were resistant. Among the patients with known outcomes, in-hospital mortality was 30/90 (33%). Age under 40 years and admission quick Sequential Organ Failure Assessment (qSOFA) score 0 were independently associated with survival.

Conclusion: Coliform BSIs of urinary origin predominated and showed substantial resistance to commonly used antibiotics. Admission qSOFA score was independently associated with survival to discharge in this cohort and may support bedside risk stratification for adults with suspected sepsis in resource-limited settings, like in Sri Lanka.

## Introduction

Bloodstream infections (BSIs) are a major cause of sepsis and in-hospital mortality worldwide, with most related deaths occurring in low- and middle-income countries (LMICs) [1]. A recent global analysis estimated that BSIs were associated with about 2.91 million deaths in 2019, with Gram-negative bacteria responsible for roughly 51% of these deaths [2]. Rudd et al. reported 48.9 million incident sepsis cases and 11.0 million sepsis-related deaths in 2017, using Global Burden of Disease data [3]. Early recognition and timely management of sepsis are crucial for improving patient outcomes and preventing progression to organ failure and death [1].

In many LMIC settings, most patients present outside intensive care units due to limited critical care resources. Simple bedside tools for risk assessment are therefore utmost important. To support early risk assessment, clinicians can use the quick Sequential Organ Failure Assessment (qSOFA) score as a simple bedside screening tool to identify patients at risk of developing sepsis. qSOFA assigns 1 point each for systolic blood pressure of 100 mmHg or less, respiratory rate of 22 breaths/minute or more, and altered mentation, for a total score of 0-3 [4,5]. Rudd et al. found, in a pooled analysis of nine cohorts from 10 low- and middle-income countries, that higher qSOFA scores were associated with substantially increased odds of in-hospital death, although discrimination varied across hospitals and regions [6]. Similarly, a recent study conducted at a tertiary care hospital in Sri Lanka involving 387 adults with sepsis reported a hospital mortality rate of 9.6%, and a higher qSOFA score was strongly associated with mortality [7].

Like many South Asian countries, Sri Lanka faces a substantial burden of sepsis and antimicrobial resistance. However, contemporary data describing the microbiology of BSIs and their clinical severity at presentation are limited. Previous work has reported on sepsis causes, outcomes, and adherence to guidelines at tertiary care hospitals, as well as antibiotic susceptibility patterns among patients with suspected sepsis [7,8]. However, there are few data specifically focusing on adults with clinically significant blood culture-positive BSIs, integrating infection patterns, illness severity at presentation as assessed by qSOFA, and detailed antibiotic susceptibility profiles within a single tertiary care setting.

The objective of this study was to describe infection patterns, antimicrobial susceptibility profiles, and factors associated with outcome among patients with positive blood cultures at Teaching Hospital Batticaloa (THB), Sri Lanka.

## Materials and methods

Study population, sampling, and sample size

This study was a retrospective analysis of 100 adults aged 18 years or older with clinically significant positive blood cultures who were admitted to the medical wards of THB from January 1 to June 30, 2022. Cases were identified from microbiology laboratory records and corresponding bed head tickets (BHTs). All eligible cases available during the study period were included. Blood cultures considered likely contaminants were excluded after review of organism type, clinical context, and treating team documentation.

Data collection

This study included positive blood cultures from adult patients admitted to the medical wards of THB during the study period. A clinically significant positive blood culture was defined as isolation of a pathogenic organism considered consistent with the patient’s clinical presentation and managed as BSI by the clinical team. The qSOFA score was calculated using the admission blood pressure, respiratory rate, and mental status recorded in the BHTs. For analysis, qSOFA was categorized as 0 vs. 1 or more. Demographic, clinical, microbiological, and outcome data were extracted from patient records using a data extraction form. Microbiological and antimicrobial susceptibility data were extracted from routine laboratory reports. As not every isolate was tested against every antibiotic, the number tested for each agent is shown. Details of blood culture collection, including the number of sets and timing, were not consistently available in the retrospective records.

Statistical analysis

Data analysis was done using the Statistical Package for Social Sciences version 26 (IBM Corp., Armonk, NY). Descriptive data were described using numbers and percentages. The outcome was defined as survival to discharge vs. in-hospital death. Survived vs. died groups were compared using the chi-square test or Fisher’s exact test for categorical variables. We used binary logistic regression to assess factors associated with survival to discharge. Variables with p < 0.20 in the bivariate analysis were included in the initial multivariable model. We report odds ratios (ORs) with 95% confidence intervals. Patients who left against medical advice were excluded from outcome regression analyses. A two-sided probability value below 0.05 (p <0.05) was considered statistically significant. Model performance was additionally assessed using omnibus model significance, the -2 log likelihood, Cox and Snell R², Nagelkerke R², and the Hosmer-Lemeshow goodness-of-fit test. Given the limited number of deaths, the final multivariable model was kept parsimonious and interpreted cautiously.

## Results

Participant characteristics

The present study recruited a sample of 100 patients. Most of them were female patients (51; 51.0%). The mean age of the patients was 53.6 years (SD = 16.31; range = 18-81) (Table 1). The majority belonged to the 45- to 64-year age category (39; 39.0%). Eighty-two (82%) of the study group had at least one comorbidity, with diabetes mellitus being the most common, occurring in 49 (49%). Hypertension and chronic kidney disease were the second and third most common comorbidities, at 41 (41%) and 20 (20%), respectively.

**Table 1:** Distribution of age in years among the participants SD: standard deviation

Characteristic	Male (n = 49)	Female (n = 51)	Total (n = 100)
Mean (SD)	54.43 (16.37)	52.88 (16.38)	53.64 (16.31)
Minimum age	15	18	15
Maximum age	81	81	81

A total of 56 patients (56.0%) had a qSOFA score of 0. Out of the total sample, 26 patients (26.0%) received inotropes. Only 13 patients (13.0%) received steroids. Most of the patients (55; 55%) were treated at the hospital for less than seven days. Overall, in-hospital mortality was 30/90 (33%) among patients with known outcomes.

Isolated organisms from blood cultures

A predominance of coliforms (54; 54.0%) was identified in BSIs, followed by *S. aureus* (19; 19.0%). Non-coliform Gram-negative bacilli (NCGNB) were found in 15 samples (15.0%). Of the coliforms (54; 54.0%), *Escherichia coli* was identified in 28 (28.0%), Klebsiella sp. in 25 (25.0%), and Proteus sp. in one (1.0%) blood culture. Notably, a high proportion of *S. aureus* isolates were methicillin-resistant (MRSA), accounting for 10/19 (52.6%) isolates, while methicillin-sensitive *S. aureus* (MSSA) accounted for 9/19 (47.4%). Acinetobacter was the main organism among NCGNB (7; 7.0%) (Table 2).

**Table 2:** Isolated organisms from blood cultures (n = 100) MRSA: methicillin-resistant *Staphylococcus aureus*; MSSA: methicillin-susceptible *Staphylococcus aureus*; NCGNB: non-coliform Gram-negative bacilli

Organism (broad category)	n (%)
Coliforms	54 (54.0)
Escherichia coli	28 (28.0)
Klebsiella spp.	25 (25.0)
Proteus spp.	1 (1.0)
Staphylococcus aureus	19 (19.0)
MRSA	10 (10.0)
MSSA	9 (9.0)
NCGNB	15 (15.0)
Acinetobacter	7 (7.0)
Pseudomonas species	2 (2.0)
Burkholderia species	2 (2.0)
Neisseria meningitidis	1 (1.0)
Other NCGNB	3 (3.0)
Multiorganism growth	2 (2.0)
Other organisms	10 (10.0)

Antimicrobial susceptibility patterns among coliform, *S. aureus,* and NCGNB isolates

Antimicrobial susceptibility testing was not performed for every antibiotic in all isolates. The number tested for each agent is indicated in Table 3.

**Table 3:** Antimicrobial susceptibility patterns among coliform, S. aureus, and NCGNB isolates NCGNB: non-coliform Gram-negative bacilli

Antibiotic	Number tested	Sensitive, n (%)
Coliform isolates (n = 54)		
Amikacin	35	34 (97.1)
Imipenem	31	30 (96.8)
Meropenem	31	30 (96.8)
Gentamicin	33	28 (84.8)
Ciprofloxacin	46	28 (60.9)
Co-amoxiclav	42	27 (64.3)
Cefepime	39	19 (48.7)
Ceftriaxone	42	19 (45.2)
Ampicillin	12	2 (16.7)
Trimethoprim-sulfamethoxazole	43	25 (58.1)
Staphylococcus aureus isolates (n = 19)		
Trimethoprim-sulfamethoxazole	18	15 (83.3)
Clindamycin	15	11 (73.3)
Flucloxacillin	7	5 (71.4)
Ciprofloxacin	6	4 (66.7)
Erythromycin	15	9 (60.0)
Cefuroxime	11	6 (54.5)
Cefepime	8	3 (37.5)
Penicillin	8	0 (0.0)
Vancomycin	6	6 (100.0)
NCGNB (n = 15)		
Ciprofloxacin	11	7 (63.6)
Amikacin	10	5 (50.0)
Imipenem	6	3 (50.0)
Meropenem	6	3 (50.0)
Ceftazidime	4	2 (50.0)
Cefepime	11	5 (45.5)
Ceftriaxone	7	3 (42.9)
Trimethoprim-sulfamethoxazole	9	4 (44.4)
Gentamicin	8	3 (37.5)

Susceptibility Patterns Among Coliform Isolates

High susceptibility was observed to aminoglycosides (amikacin and gentamicin) and carbapenems (imipenem and meropenem). Amikacin sensitivity was 97.1% (34/35). Imipenem and meropenem each showed sensitivity of 96.8% (30/31). Gentamicin sensitivity was 84.8% (28). Ciprofloxacin sensitivity was 60.9% (28). Among the cephalosporins, cefepime showed 51.3% (20) resistance, while ceftriaxone showed 54.8% (23) resistance. Ampicillin had a resistance of 83.3% (10).

Susceptibility Patterns Among S. aureus Isolates

High susceptibility was observed to vancomycin (6; 100.0%). Trimethoprim-sulfamethoxazole showed a sensitivity of 83.3% (15). Clindamycin sensitivity was 73.3% (11). Flucloxacillin sensitivity was 71.4% (5), with 28.6% (2) resistant. Ciprofloxacin sensitivity was 66.7% (4), with 33.3% (2) resistant. Lower sensitivity was observed to macrolides, cephalosporins, and penicillin. All isolates (8;100.0%) tested against penicillin were resistant.

Susceptibility Patterns Among NCGNB Isolates

Ciprofloxacin sensitivity was 63.6% (7), while Amikacin sensitivity was 50.0% (5). Imipenem and meropenem each showed sensitivity of 50.0% (3). Ceftazidime sensitivity was 50.0% (2). Cefepime showed sensitivity of 45.5% (5), while ceftriaxone showed 57.1% (4) resistance.

The focus of infection of bacteremia

In a total of 54 cases of Coliform bacteremia, urosepsis was the most common focus (28; 51.8%), followed by intra-abdominal infection (9; 16.7%). Pneumonia occurred in five patients (9.3%). In a total of 19 cases of *S. aureus* bacteremia, the most frequent focus was central line-associated BSIs, accounting for nine cases (47.4%). Pneumonia occurred in three cases (15.7%). Sepsis without a defined focus and infective endocarditis occurred in two cases each (10.5%) (Table 4).

**Table 4:** Focus of infection of coliform and S. aureus bacteremia BSIs: bloodstream infections

Focus of infection	n (%)
Coliform (n = 54)	
Urosepsis	28 (51.8)
Intra-abdominal infection	9 (16.7)
Pneumonia	5 (9.3)
BSIs due to central vascular line	4 (7.4)
Sepsis without a focus	4 (7.4)
Skin and soft tissue infections	4 (7.4)
Staphylococcus aureus (n = 19)	
Central line-associated bloodstream infection	9 (47.4)
Pneumonia	3 (15.7)
Sepsis without a focus	2 (10.5)
Infective endocarditis	2 (10.5)
Secondary bacterial infection after dengue	1 (5.3)
Pyomyositis	1 (5.3)
Pyelonephritis	1 (5.3)

Association between outcome and selected variables

Age under 40 years had higher survival to discharge than age 40 years or above (OR = 6.49; p=0.008). There was no statistically significant difference in survival by gender or comorbidity category. A qSOFA score of 0 showed higher survival than scores 1 or more (OR = 3.21; p = 0.011). In contrast, the use of inotropes and steroids was strongly linked with death: OR = 0.03 (p < 0.001) and OR = 0.07 (p < 0.001), respectively. A hospital stay of seven days or less was associated with lower odds of survival than a stay over seven days (OR = 0.35; p = 0.025) (Table 5).

**Table 5:** Association between outcome and selected variables (n = 90) The outcomes of 10 patients were not available because they left against medical advice p values were derived from chi-square test or Fisher’s exact test, as indicated. Odds ratios are shown for comparison with the regression analysis ^*^Fisher’s exact test CI: confidence interval; qSOFA: quick sequential organ failure assessment

Variable	Outcome		Odds ratio (95% CI)	p value
	Survive, n (%)	Died, n (%)		
Age				
<40 years	19 (90.5)	2 (9.5)	6.49 (1.40-30.09)	0.008^*^
≥40 years (reference)	41 (59.4)	28 (40.6)		
Gender				
Female	32 (71.1)	13 (28.9)	1.50 (0.62-3.61)	0.371
Male (reference)	28 (62.2)	17 (37.8)		
Comorbidity				
Present	49 (67.1)	24 (32.9)	1.11 (0.37-3.37)	0.849
Absent (reference)	11 (64.7)	6 (35.3)		
qSOFA score				
0	39 (78.0)	11 (22.0)	3.21 (1.29-7.99)	0.011
≥1 (reference)	21 (52.5)	19 (47.5)		
Inotropes				
Given	3 (13.6)	19 (86.4)	0.030 (0.01-0.12)	<0.001^*^
Not given (reference)	57 (83.8)	11 (16.2)		
Steroids				
Given	2 (16.7)	10 (83.3)	0.07 (0.01-0.34)	<0.001
Not given (reference)	58 (74.4)	20 (25.6)		
Hospital stay				
≤7 days	27 (56.3)	21 (43.8)	0.35 (0.14-0.89)	0.025
>7 days (reference)	33 (78.6)	9 (21.4)		

Multivariable analysis of factors associated with outcomes

Variables with p < 0.20 in the bivariate analysis were selected for initial model building, as shown in Table 4. The obtained model was further evaluated by deleting and readding variables, and the final parsimonious model included age and qSOFA score. The final binary logistic regression model was significant overall on the omnibus test (χ² = 13.558, df = 2, p = 0.001). The model summary showed a -2 log likelihood of 101.015, Cox and Snell R² of 0.140, and Nagelkerke R² of 0.194. Calibration and goodness of fit were assessed using the Hosmer-Lemeshow test, which was nonsignificant (χ² = 0.053, df = 2, p = 0.974), suggesting no evidence of poor fit in this sample. In the final model, age under 40 years and an admission qSOFA score of 0 remained independently associated with survival to discharge (Table 6).

**Table 6:** Multivariable analysis of factors associated with outcome (n = 90) The outcomes of 10 patients were not available because they left against medical advice OR: odds ratio; CI: confidence interval; qSOFA: quick sequential organ failure assessment

Predictor	Crude OR (95% CI)	p value	Adjusted OR (95% CI)	p value
Age				
<40 years	6.49 (1.40-30.09)	0.008	6.03 (1.27-28.67)	0.024
≥40 years (reference)				
qSOFA score				
0	3.21 (1.29-7.99)	0.011	3.01 (1.17-7.74)	0.023

Model summary

The results of the statistical analysis are as follows: Omnibus test: χ² = 13.558, df = 2, p = 0.001; -2 log likelihood = 101.015; Cox and Snell R² = 0.140; Nagelkerke R² = 0.194; and Hosmer-Lemeshow test (χ² = 0.053, df = 2, p = 0.974). The adjusted analysis should be interpreted as showing independent associations with survival to discharge in this cohort, not as external validation of a predictive model.

## Discussion

This study describes the infection patterns and antimicrobial susceptibility among patients with culture-positive BSIs. Gram-negative organisms, mainly coliforms, predominated, and mortality was about one-third. Gram-negative BSIs in healthcare settings have also been associated with increased mortality in other cohorts [9]. These findings align with the global picture where BSIs and sepsis cause substantial morbidity and death, particularly in LMICs [1-3].

This study has several strengths. It addresses a clinically important problem in a setting with limited published local data. It provides linked clinical and microbiological information on BSIs, including organism distribution, likely infection focus, and antibiotic susceptibility patterns. It also evaluates admission qSOFA as a simple bedside prognostic tool and uses logistic regression to examine factors associated with in-hospital outcome.

Recent studies conducted in Sri Lanka have reported sepsis and urinary tract infections caused largely by Enterobacteriaceae and have also highlighted urinary sources as common causes of bacteremia [8,10,11]. The high proportion of central line-associated Staphylococcus aureus bacteremia in the present study underscores the importance of vascular devices as a source of hospital-acquired infections, consistent with global data on device-related sepsis [12].

Coliforms remained highly susceptible to amikacin and carbapenems, while showing poor susceptibility to the third- and fourth-generation cephalosporins and ampicillin. Similar profiles have been described in previous Sri Lankan studies [13]. These studies highlighted resistance to cephalosporins exceeded 50% and susceptibility to amikacin and carbapenems remained high [10,11,13]. The *S. aureus* susceptibility profile is also in line with national data. Approximately half of the *S. aureus* isolates were methicillin-resistant, and all isolates tested against vancomycin were susceptible. Previous local studies have reported similar MRSA proportions and preserved activity of vancomycin and other glycopeptides [14,15]. The combination of frequent MRSA bacteremia and central line-related infection in this cohort stresses the need for strict catheter insertion and maintenance practices.

Younger age was independently associated with survival, consistent with global sepsis analyses that report rising mortality with advancing age and comorbidity [3,16]. In bivariate analysis, inotrope and steroid use were strongly associated with death, but these variables were not retained in the final multivariable model. Therefore, they should be interpreted only as crude associations in this cohort, and confounding by indication was not formally tested.

An admission qSOFA score of 0 was independently associated with higher odds of survival to discharge in this cohort. However, given the modest sample size, limited number of outcome events, and lack of external validation, this finding should be interpreted cautiously as a cohort-specific association rather than a fully validated predictive model. This supports the finding of previous local studies and other LMIC settings where higher qSOFA scores correlated with increased in-hospital mortality among adults with suspected sepsis [6,7]. Although qSOFA is commonly discussed using a threshold of 2 or more, our analysis examined qSOFA of 0 vs. 1 or more to distinguish patients with no abnormal qSOFA components at admission from those with at least one abnormal component. Systematic reviews indicate that qSOFA has only modest discrimination in some populations, so it should support rather than replace clinical judgment [4,17]. In this context, routine calculation of qSOFA from admission vital signs offers a practical way to flag higher risk patients for closer monitoring and timely escalation in resource-limited settings. Therefore, the qSOFA findings in this study should be interpreted as supportive cohort-specific risk-stratification data rather than as directly comparable validation against conventional qSOFA thresholds.

This study has several limitations. First, it is a single-center retrospective analysis with a relatively small sample, which restricts generalizability. Second, the use of convenience sampling and routine clinical documentation may lead to selection bias and misclassification of qSOFA components or comorbidities. Third, the distinction between true BSI and contamination is an inherent limitation of retrospective studies. Fourth, some antibiotics were tested only on a subset of isolates, and molecular typing to confirm extended-spectrum beta-lactamase or carbapenemase production was unavailable. Fifth, the qSOFA categorization used in this study, 0 vs. 1 or more, differs from the conventional threshold used in many validation studies, so comparisons across studies should be made cautiously. Sixth, the number of deaths was limited for multivariable modeling, so the final model should be interpreted cautiously. Finally, the duration of hospital stay may have been influenced by the outcome itself and, therefore, should be interpreted cautiously as a clinical correlate rather than a baseline predictor.

## Conclusions

Our study provides recent linked clinical and microbiological data on culture-proven BSIs from a region of Sri Lanka with limited published evidence. Coliform bacteremia of urinary origin, often resistant to commonly used antibiotics, accounts for most BSIs in this hospital. An admission qSOFA score of 0 was associated with survival to discharge and may support early bedside risk stratification in adults with suspected sepsis. We recommend future multicenter studies with larger sample sizes to improve the generalizability of the data.
